# *KRAS* Mutant Allele Fraction in Circulating Cell-Free DNA Correlates With Clinical Stage in Pancreatic Cancer Patients

**DOI:** 10.3389/fonc.2019.01295

**Published:** 2019-11-29

**Authors:** Zhe-Ying Wang, Xiao-Qing Ding, Hui Zhu, Rui-Xian Wang, Xiao-Rong Pan, Jian-Hua Tong

**Affiliations:** Faculty of Medical Laboratory Science and Central Laboratory, Ruijin Hospital, Shanghai Jiao Tong University School of Medicine, Shanghai, China

**Keywords:** circulating tumor DNA, *KRAS* mutation, pancreatic cancer, liquid biopsy, droplet digital PCR

## Abstract

**Background:** The research on circulating tumor DNA (ctDNA) in pancreatic cancer (PC) has emerged recently. Although the detection rate of the *KRAS* mutation in ctDNA was relatively consistent with that in tumor tissue, whether the *KRAS* mutant allele fraction (MAF) differed was still not reported. So far, the clinical application of ctDNA detection in PC remains inconclusive.

**Methods:** Plasma samples were collected from 110 PC and 52 pancreatic benign (PB) disease patients. The detection of *KRAS* mutation in ctDNA was performed using droplet digital PCR and compared with that in matched tumor tissue. We assessed the utility of *KRAS* MAFs in ctDNA and tissue for pancreatic malignancy assessment.

**Results:** We found that *KRAS* MAF in ctDNA of PC patients was higher than that of PB patients, and was obviously associated with tumor staging and distant metastasis. However, *KRAS* MAF in ctDNA was significantly different from that in matched tissue. *KRAS* MAF in tumor tissue had no significant correlation with the clinical status. In addition, a ROC curve analysis revealed that mutant *KRAS* ctDNA combined with CA19-9 could increase the sensitivity rate of early-stage PC prediction, compared with CA19-9 test alone.

**Conclusion:** The MAF of *KRAS* in ctDNA was related to the clinical stage of PC (*p* = 0.001). Mutant *KRAS* ctDNA could improve the sensitivity in early diagnosis of PC as a complement to CA19-9. Our study suggested that *KRAS* mutation in ctDNA could be a valuable circulating biomarker for the malignancy assessment in PC.

## Introduction

Pancreatic cancer (PC) is one of the most lethal malignancies in the world, with a 5-year overall survival rate of <6% (1, 2). The poor prognosis is partly due to the fact that PC is usually diagnosed at advanced stages and resistant to therapy (3). Therefore, the opportunity for improving patient prognosis lies in earlier diagnosis and monitoring of cancer progress and recurrence. Usually, carbohydrate antigen 19-9 (CA19-9) is a routine tumor biomarker used in the diagnosis and management of patients with PC. However, the application of CA19-9 is limited due to its low specificity in non-malignant situations, such as cholestasis or diabetes mellitus (4, 5).

As is well-known, the occurrence and the development of cancer results from the accumulation of genetic aberrations in DNA. Therefore, these genetic alterations could serve as specific biomarkers for cancer patients. It is reported that *KRAS* is one of the most frequently mutated genes in PC, with a detection rate in tumor tissue ranging from 75 to 95% (6). However, tumor tissue sampling is usually not convenient, particularly for patients who cannot undergo surgical resection. Recently, it has been shown that mutant DNA within tumor cells can be released into blood, and the research on circulating tumor DNA (ctDNA) in plasma has evolved as an exciting field in oncology (7–10). Besides the ease of blood sample acquirement, it is more likely that ctDNA could reflect the overall tumor burden in comparison with tumor tissue. *KRAS* mutations in ctDNA have been previously studied in the context of PC, with a detection rate ranging from 41.3 to 75% (11–14). Although the presence of *KRAS* mutation in ctDNA was relatively consistent with that in tumor tissue of the same person, whether the mutant allele fraction (MAF) of *KRAS* in ctDNA and tumor tissue differed was still not reported. In addition, since the content of cell-free DNA (cfDNA) in the circulation is too low, the application of ctDNA detection in diagnosis and disease evaluation of PC remains inconclusive. With technologic advances, the methods for quantitative detection of ctDNA, including droplet digital PCR (ddPCR) and optimized next generation sequencing (NGS) strategies, have rapidly developed. Since ddPCR affords a very high level of sensitivity and absolute quantification for the target molecules, it is considered as one of the most important methods for mutant ctDNA detection (15).

In this study, we detected the common hot spot mutations in *KRAS* of ctDNA using ddPCR and compared it with that of tissue samples using standard clinical testing. In addition, we analyzed the utility of *KRAS* mutation in ctDNA and paired tissue samples of PC and pancreatic benign disease (PB) patients for malignancy assessment.

## Materials and Methods

### Plasma Samples

Whole blood samples from 110 PC and 52 PB patients were obtained prior to treatment in Ruijin Hospital (Shanghai, China). One to four milliliter of peripheral blood was collected in EDTA tubes (BD) and centrifuged at 1,900 × g for 10 min at room temperature and then at 16,000 × g for 10 min at 4°C. Isolated plasma samples were stored at −80°C until needed.

### Cell Culture

Cell lines (PANC-1, BxPC-3, and HCT116) were obtained from Shanghai Institute of Digestive Surgery (Shanghai, China). Briefly, PANC-1, BxPC-3, and HCT116 cells were cultured in DMEM, RPMI 1640, and McCoy's 5A (Gibco) with 10% fetal bovine serum (Sigma), respectively. Cell culture was performed at 37°C in a 5% CO_2_ atmosphere. Before supernatant cfDNA isolation, cells were grown at an initial density of 2.5 × 10^5^/ml for 48 h. Then the cell culture supernatants were collected and cleared by two-step centrifugation as described for plasma samples.

### cfDNA Isolation and Quantification

Total cfDNA was extracted from plasma or cell culture supernatant with use of the QIAamp ccfDNA/RNA Kit (Qiagen) according to the manufacturer's protocol, then quantified using the Qubit 3.0 fluorometer (Thermo Fisher). For patient samples, the concentration of cfDNA was indicated as nanograms per milliliter of plasma.

### Primers and Probes

The primers (Beijing Genomics Institute) used to amplify a segment in exon 2 of *KRAS* gene (78-bp amplicon) were as follows: Forward primer, 5′-GCCTGCTGAAAATGACTGAAT-3′; Reverse primer, 5′-GCTGTATCGTCAAGGCACTCT-3′. Multiple hotspot mutations within codon 12 and codon 13 of *KRAS* gene exon 2 were detected with a pair of drop-off and reference probes according to the method described by Decraene et al. (16). A drop-off probe with a 5′ fluorophore and a 3′ non-fluorescent quencher (NFQ) was designed covering hotspot mutation regions in exon 2 of the wild-type *KRAS* gene. A reference probe was designed upstream of the drop-off probe over a non-mutated region in the same amplicon, with a 5' fluorophore and a 3′ NFQ. The sequences of these two probes (Life Technologies) were 5′-(6-FAM)-CTACGCCACCAGCT-(MGB NFQ)-3′ and 5′-(VIC)-CAACTACCACAAGTTT-(MGB NFQ)-3′, respectively (Supplementary Figure 1A). Thus, wild-type *KRAS* molecules were double fluorescence signal positive, while mutant molecules sub-optimally hybridized to the drop-off probe due to a mismatch and presented a decrease in the drop-off probe signal.

### Detection of *KRAS* Mutations in cfDNA

The ddPCR platform (Qx200 ddPCR system, Bio-Rad) was used for the detection of *KRAS* mutations in cfDNA as per the manufacturer's instruction. Twenty microliter ddPCR reaction solutions were prepared with dUTP-free Supermix for probes (Bio-Rad), 900 nM of primers, 250 nM of hydrolysis probes and at least 0.5 ng of cfDNA. The amplification was performed under the following conditions: 95°C for 10 min, 40 cycles of (94°C for 30 s, 60°C for 60 s), 98°C for 10 min. The results were analyzed by the Quanta-Soft Analysis Pro software (Bio-Rad).

### Calculation of the LOB and LOD

The limit of blank (LOB) and the limit of detection (LOD) for the ddPCR analysis were measured according to the Clinical and Laboratory Standards Institute guidelines EP17.2 and relevant reports (16–18). CfDNA templates extracted from BxPC-3 cells with wild-type *KRAS* were used as blank samples. Forty replicates of blank samples were detected by the ddPCR assay to calculate the LOB, which corresponds to the 95th percentile of distribution of the blank values. For LOD, cfDNA templates extracted from PANC-1 cells with mutant *KRAS* (c.35G>A, p.G12D) and HCT116 cells with mutant *KRAS* (c.38G>A, p.G13D) were diluted with the wild-type cfDNA to the expected MAFs (5, 2.5, 1.25, 0.63, 0.31, 0.16, and 0.08%), respectively. Then a series of cfDNA samples with different MAFs were detected by the ddPCR assay and repeated for at least 6 independent experiments. After consideration on the results of G12D and G13D, the LOD was defined as the MAF value in which the 95% confidence interval of all replicate detections presented values above the LOB.

### Detection of *KRAS* Status in Tissue Samples

Matched formalin fixed paraffin embedded (FFPE) tissue specimens were obtained according to the clinical histopathologic results. DNA was extracted using Qiagen GeneRead DNA FFPE Kit (Qiagen) as per the instruction of the manufacturer. The *KRAS* status was determined using NGS on Illumina MiSeq System (Illumina).

### Statistical Analyses

Statistical analyses were performed using the IBM SPSS Version 23 software (IBM Corporation, Armonk, NY, USA). Continuous data were compared using the Wilcoxon and Kruskal-Wallis tests. The correlation analysis was performed using the Spearman rank test. The accuracy of the circulating biomarkers was analyzed using a receiver operating characteristic (ROC) curve. A *p* < 0.05 was considered as significant.

## Results

### Patient Characteristics

In total, 162 patients with pancreatic space occupying lesions (110 malignant and 52 benign) were included in this study. The characteristics of the patients are listed in Table 1. The PC patients included 99 pancreatic ductal adenocarcinoma and 11 other malignant tumors. For the PB patients, the most common diseases diagnosed were serous cystadenoma (*n* = 14, 26.9%), pseudocyst (*n* = 14, 26.9%), intraductal papillary mucinoma (*n* = 11, 21.2%), and mucinous cystadenoma (*n* = 9, 17.3%). The median CA19-9 concentration in PC was higher than that in PB (175.2 U/ml vs. 9.0 U/ml, *p* < 0.001).

**Table 1:** Patient characteristics.

**Groups**		**Pancreatic cancer**	**Pancreatic benign disease**
Patient number (n)		110	52
Age, mean (range) (year)		65 (40–91)	55 (22–81)
Gender (percentage)	Female	47 (42.7%)	27 (51.9%)
	Male	63 (57.3%)	25 (48.1%)
Clinical stage (percentage)	I	31 (27.4%)	–
	II	24 (21.2%)	–
	III	32 (29.1%)	–
	IV	20 (18.2%)	–
CA19-9, median (range) (U/ml)		175.2 (0.8–20190.0)	9.0 (0.80–317.40)
cfDNA, median (range) (ng/ml plasma)		8.38 (0.55–95.40)	7.83 (0.62–63.93)

*cfDNA, cell-free DNA*.

### *KRAS* Mutation Status in cfDNA and Pancreatic Tissue

**Figure 1:**
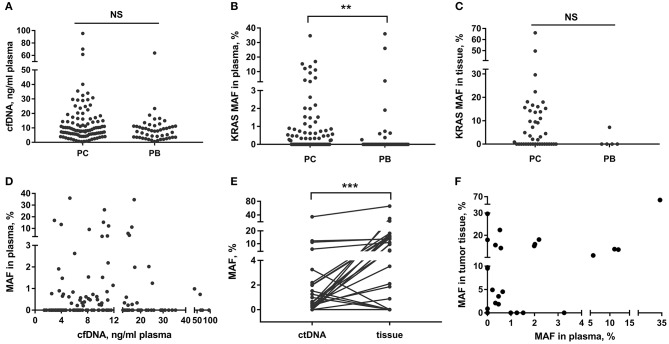
cfDNA content and *KRAS* mutation in PC and PB patients. **(A–C)** Detection of total cfDNA amount and *KRAS* MAF in ctDNA and tissue in PC and PB diseases. **(D)** Correlation between *KRAS* MAF in ctDNA and cfDNA concentration. **(E,F)** Comparison of *KRAS* MAF in ctDNA and matched tissue. ^**^p < 0.01; ^***^p < 0.001; NS, not significant.

In this study, we detected that the LOB of the ddPCR analysis on the mutant *KRAS* ctDNA was 0.10%. The LODs were 0.16% for 2 ng and 5 ng of cfDNA templates, 0.63% for 1 ng, and 1.25% for 0.5 ng (Supplementary Figures 1B,C). The cfDNA content did not show obvious differences in the PC and PB groups (Figure 1A, *p* = 0.066). Then the ddPCR detection was performed in 138 (85%) cases with adequate cfDNA amount in the study cohort. The detection rate of mutant *KRAS* ctDNA were 47.4% (45/95) in PC and 20.9% (9/43) in PB, respectively. *KRAS* mutation in tissue was present in 58.1% (25/43) of malignant samples and 20% (1/5) of benign samples, respectively (Figure 1C). *KRAS* MAFs in ctDNA of PC were much higher than that of PB (Figure 1B, *p* = 0.007). In addition, we demonstrated there was no significant correlation between cfDNA concentration and *KRAS* MAF in ctDNA (Figure 1D, *p* = 0.663, *r*^2^ = 0.001).

### Discordance of MAFs Between ctDNA and Matched Tissue

We also compared the *KRAS* mutation status in ctDNA and matched tissue in 35 patients (34 PC and 1 PB). The blood and tissue samples were collected at the same time. The concordance rate of *KRAS* mutation status (mutant or wild-type) in ctDNA and tissue was 74.3% (26/35) (Table 2). However, the MAF of *KRAS* in ctDNA was significantly different from that in tumor tissue of the same person (Figure 1E, *p* < 0.001). The median MAFs of *KRAS* in ctDNA and tumor tissue were 0.34% (range: 0–34.71%) and 2.81% (range: 0–66.10%), respectively. In addition, the correlation between *KRAS* MAFs in ctDNA and tumor tissue was poor (Figure 1F, *p* = 0.007, *r*^2^ = 0.200).

**Table 2:** Results of *KRAS* mutation detection in ctDNA and matched tissue.

**Groups**		**PC or PB tissue**		
		***KRAS* mutant**	***KRAS* wild-type**	**Total**
ctDNA	*KRAS* mutant	15	4	19
	*KRAS* wild-type	5	11	16
	Total	20	15	35

*ctDNA, circulating tumor DNA*.

### *KRAS* MAF in ctDNA in Relation to Clinical Stages of PC

**Figure 2:**
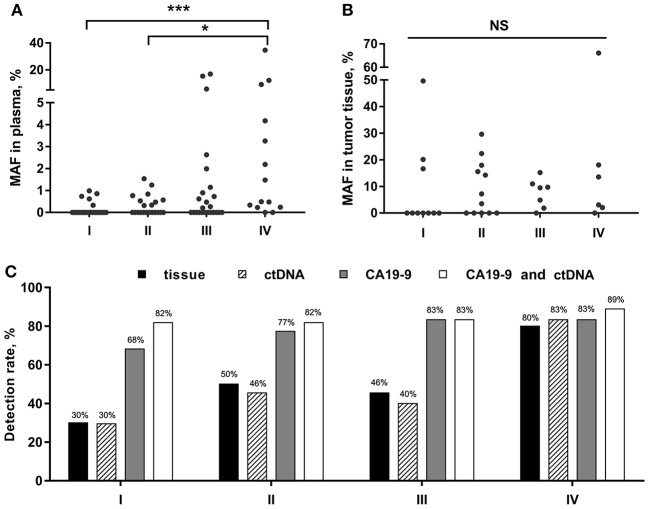
*KRAS* MAFs and the detection rate for PC in different clinical stages. **(A,B)** Correlation of *KRAS* MAF in ctDNA and tumor tissue with PC stages. **(C)** PC detection rate by tissue, ctDNA, CA19-9, and the combined assay of ctDNA and CA19-9. ^*^p < 0.05; ^***^p < 0.001; NS, not significant.

We then analyzed the correlation between *KRAS* MAF in ctDNA or tumor tissue and clinical characteristics of PC patients. The *KRAS* MAF in ctDNA was obviously associated with clinical staging (*p* = 0.001) and the presence of distant metastasis (*p* < 0.001) in PC (Table 3, Figure 2A). Patients in stage IV had significantly greater *KRAS* MAF in ctDNA than patients in stage I/II (*p* < 0.001 and 0.031). *KRAS* MAFs in patients of stage I and II were all below 2%. In comparison with *KRAS* MAF in ctDNA, there was no statistically significant difference in *KRAS* MAF in tumor tissue, when the PC patients were stratified with either clinical staging or the presence of distant metastasis (Table 3, Figure 2B, *p* = 0.498 and 0.370).

**Table 3:** Correlation of *KRAS* MAFs with clinical status of pancreatic cancer patients.

**Clinical status**		***KRAS* MAF in ctDNA, median (range) (%)**	***p*-value**	***KRAS* MAF in tumor tissue, median (range) (%)**	***p*-value**
Age (year)	40–59	0 (0–16.98)	0.803	3.10 (0–29.64)	0.080
	60–69	0 (0–15.38)		0 (0–22.39)	
	70–91	0.22 (0–34.71)		13.61 (0–66.10)	
Gender	Female	0 (0–16.98)	0.783	1.05 (0–29.64)	0.565
	Male	0 (0–34.71)		3.52 (0–66.10)	
Diagnosis	PDAC	0.23 (0–16.98)	0.733	3.31 (0–66.10)	0.420
	Others	0 (0–12.18)		0 (0–13.61)	
Tumor location	Head	0 (0–34.71)	0.236	0 (0–66.10)	0.066
	Body/tail	0.23 (0–16.98)		8.18 (0–29.64)	
AJCC stage	I	0 (0–0.99)	0.001^**^	0 (0–49.67)	0.498
	II	0 (0–13.45)		5.38 (0–29.64)	
	III	0 (0–16.98)		4.91 (0–16.02)	
	IV	1.07 (0–34.71)		3.82 (0–66.10)	
Tumor size (cm)	0–2	0 (0–1.99)	0.376	0 (0–15.24)	0.178
	2.1–3	0 (0–12.18)		0.44 (0–17.96)	
	3.1–4	0 (0–0.86)		10.79 (0–49.67)	
	4.1–15	0.22 (0–16.98)		2.60 (0–14.27)	
Lymphatic metastasis	No	0 (0–16.98)	0.139	0.44 (0–49.67)	0.088
	Yes	0.33 (0–9.17)		9.51 (0–29.64)	
Distant metastasis	No	0 (0–16.98)	<0.001^***^	1.86 (0–49.67)	0.370
	Yes	1.07 (0–34.71)		3.82 (0–66.10)	

*MAF, mutant allele fraction; ctDNA, circulating tumor DNA; PDAC, pancreatic ductal adenocarcinoma; AJCC, American Joint Committee on Cancer*.

** *p < 0.01*,

*** *p < 0.001*.

### *KRAS* Mutation in ctDNA and Tissue as Biomarkers of Malignancy Prediction

Furtherly, we analyzed the diagnostic utility of *KRAS* mutation in ctDNA, CA19-9, and *KRAS* mutation in tissue as PC biomarkers (Table 4). ROC curve analysis showed that the overall sensitivity and specificity of mutant *KRAS* ctDNA detection were 47 and 80%, respectively. According to the cancer stages, the sensitivity rates of the ctDNA detection were 30, 46, 40, and 83%, respectively (Figure 2C). In order to improve the sensitivity of the circulating biomarker test, the combination of mutant *KRAS* ctDNA with CA19-9 was employed. In comparison with mutant *KRAS* ctDNA detection or CA19-9 test alone, the overall sensitivity of the combined assay was increased to 82% at the specificity of 81% (Table 4). The sensitivity rates of the combined assay in cancer stage I–IV were improved to 82, 82, 83, and 89%, respectively (Figure 2C).

**Table 4:** Performance of circulating biomarkers in comparison with *KRAS* mutation in tissue for malignancy prediction.

**Tests**	**Patient number (*n*)**	**Sensitivity (%)**	**Specificity (%)**
*KRAS* MAF in PB or PC tissue	43	42	100
*KRAS* MAF in ctDNA	93	47	80
CA19-9	100	76	85
Combination assay of ctDNA and CA19-9	93	82	81

*MAF, mutant allele fraction; ctDNA, circulating tumor DNA*.

## Discussion

In recent years, ctDNA has gained substantial attention in the field of clinical oncology (19). The mutations of *KRAS* were detected in numerous tumors. In PC, the presence of *KRAS* mutation in ctDNA and tumor tissue was relatively consistent and the concordance rate was around 54–91% (20–24). However, the comparison between the MAF of *KRAS* in ctDNA and tumor tissue was rarely reported. In this study, we compared the detection rate of *KRAS* mutation in ctDNA with that in pancreatic tissue and found their concordance rate was 74.3%. Furthermore, we analyzed the MAF of *KRAS* in PC, and revealed for the first time that the *KRAS* MAF in ctDNA was significantly different from that in matched tumor tissue. In contrast to *KRAS* MAF in ctDNA, *KRAS* MAF in tumor tissue was not obviously different in PC patients stratified with either tumor staging or distant metastasis. Some studies also reported that the prognostic utility of *KRAS* mutation in ctDNA and in tumor tissue was discordant. Mutant *KRAS* ctDNA was considered to be a better prognostic biomarker for PC, whereas *KRAS* MAF in tumor tissue was not (14, 25). These findings might be due to the fact that a part of tumor tissue could only provide limited information. As we know, tumor tissue is very heterogeneous and will inevitably be contaminated with non-tumor cells that will skew the MAF data results (24, 26). Partial tissue sampling may lead to false negative results of mutation detection. However, ctDNA could better reflect the overall tumor burden in PC patients. The detection of ctDNA could improve the mutation detection rate as a good supplement to tumor tissue.

Current studies on PC showed that the total amount of circulating cfDNA and *KRAS* mutations in ctDNA might be associated with pancreatic tumor burden, but the conclusions remained inconsistent. For example, Wei et al. demonstrated that the total cfDNA level of patients with advanced PC was higher than that of healthy controls. The PC patients in stage IV had greater *KRAS* MAFs in ctDNA than patients in stage III (27). Bernard et al. found that the *KRAS* MAFs in ctDNA were significantly different in patients with pancreatic cysts, localized PC, and metastatic PC (20). On the other hand, some studies reported that mutant *KRAS* ctDNA or total cfDNA were statistically irrelevant with respect to tumor staging (28, 29). In the present study, we detected that PC patients had higher levels of *KRAS* MAF in ctDNA in comparison with PB patients. Moreover, *KRAS* MAF in ctDNA was obviously associated with tumor staging and distant metastasis in PC patients. PC patients in stage IV had greater *KRAS* MAF in ctDNA than patients in stage I/II (*p* < 0.001 and 0.031), while there was no significant difference between KRAS MAFs in ctDNA in stage III and stage IV patients (*p* = 0.209). Our results showed that *KRAS* MAF in ctDNA might be a good indicator of tumor burden in PC. In addition, unlike the mutant ctDNA, the total cfDNA concentration showed no significant difference in PB and different stages of PC patients. Similarly, there was no statistically significant correlation between total cfDNA concentration and *KRAS* MAF in ctDNA. Of note, we also found that the mutant *KRAS* ctDNA was detected in 20.9% (9/43) of PB patients. According to the literature, genetic mutations such as *KRAS* mutations may occur in healthy people and patients with benign diseases (30, 31). For these people, whether these genetic mutations were pre-cancerous or normal events needs to be explored through large-scale and long-term experiments.

Though the *KRAS* mutation in ctDNA was presumed to be a promising diagnostic biomarker of PC, it could only be detected in nearly half of PC patients, with detection rates of 24.4–34% in early stage patients and 53–74% in advanced patients (12, 20, 29). Therefore, the application of mutant *KRAS* ctDNA as a predictor of PC was limited because of its relatively low sensitivity. CA19-9 is routinely detected in the diagnosis and management of patients with PC. However, the well-known limitation of CA19-9 is its low specificity in non-malignant situations such as cholestasis (32). The combined assay of mutant ctDNA and protein biomarkers such as CA19-9 was performed for PC diagnosis in several studies, showing higher sensitivity than mutant ctDNA detection alone (33–35). In this study, we analyzed the sensitivity rates of *KRAS* mutation in ctDNA and tumor tissue for PC prediction and found that neither was satisfactory. In the combined assay of mutant *KRAS* ctDNA and CA19-9, the sensitivity for PC diagnosis was obviously improved especially in the early cancer stages. Compared with each single test, the combined sensitivity was raised to 82% from 30% (ctDNA) and 68% (CA19-9) in stage I and 82% from 46% (ctDNA) and 77% (CA19-9) in stage II, respectively. Therefore, mutant *KRAS* ctDNA might be helpful in early diagnosis of PC as a complement to CA19-9.

In order to better apply these study results in clinical practice, it is essential to establish a standardization to compare the data obtained from different investigators (36). The LOD is the value that can be detected in samples with minimum analytes, which is used to reflect the sensitivity of the detection. In theory, the quantity of DNA templates in PCR assay will influence the LOD value. According to our data, we showed that the LOD value of the ddPCR detection increased while the amount of cfDNA templates decreased. Thus, when the LOD value of the detection was provided, the amount of DNA template used should be indicated. Only MAF value above the corresponding LOD could be defined as positive result.

In conclusion, our study showed that *KRAS* MAF in ctDNA differed from that in tumor tissue in PC. The mutant *KRAS* ctDNA was found to be related with the clinical stage of PC patients, whereas *KRAS* MAF in tumor tissue was not. In addition, the combination of mutant *KRAS* ctDNA and CA19-9 could be a valuable circulating biomarker for early detection and diagnosis of PC. Nevertheless, here we only carried out a study on PC patients before treatment. The dynamic change of *KRAS* MAF in ctDNA of PC patients during treatment should be continuously detected in our next work. The clinical application of mutant *KRAS* ctDNA in the diagnosis and monitoring of PC still needs further validation by large-scale clinical studies.

## Data Availability Statement

The datasets generated for this study are available on request to the corresponding author.

## Ethics Statement

The studies involving human participants were reviewed and approved by Ruijin Hospital Ethics Committee. The patients/participants provided their written informed consent to participate in this study.

## Author Contributions

Z-YW and J-HT designed the study. Z-YW, X-QD, HZ, and R-XW conducted experiments, analyzed data, and wrote the manuscript. Z-YW, X-RP, and J-HT supervised research, interpreted data, and revised the manuscript.

### Conflict of Interest

The authors declare that the research was conducted in the absence of any commercial or financial relationships that could be construed as a potential conflict of interest.
