# Supplementary material for: KRAS Mutant Allele Fraction in Circulating Cell-Free DNA Correlates With Clinical Stage in Pancreatic Cancer Patients
Source: Front Oncol. 2019 Nov 29;9:1295. doi: 10.3389/fonc.2019.01295 (PMC6896365; doi:10.3389/fonc.2019.01295)
Supplement: Supplementary file 1 [file Table_1.DOCX]

Supplementary Material

# Supplementary Figure

**
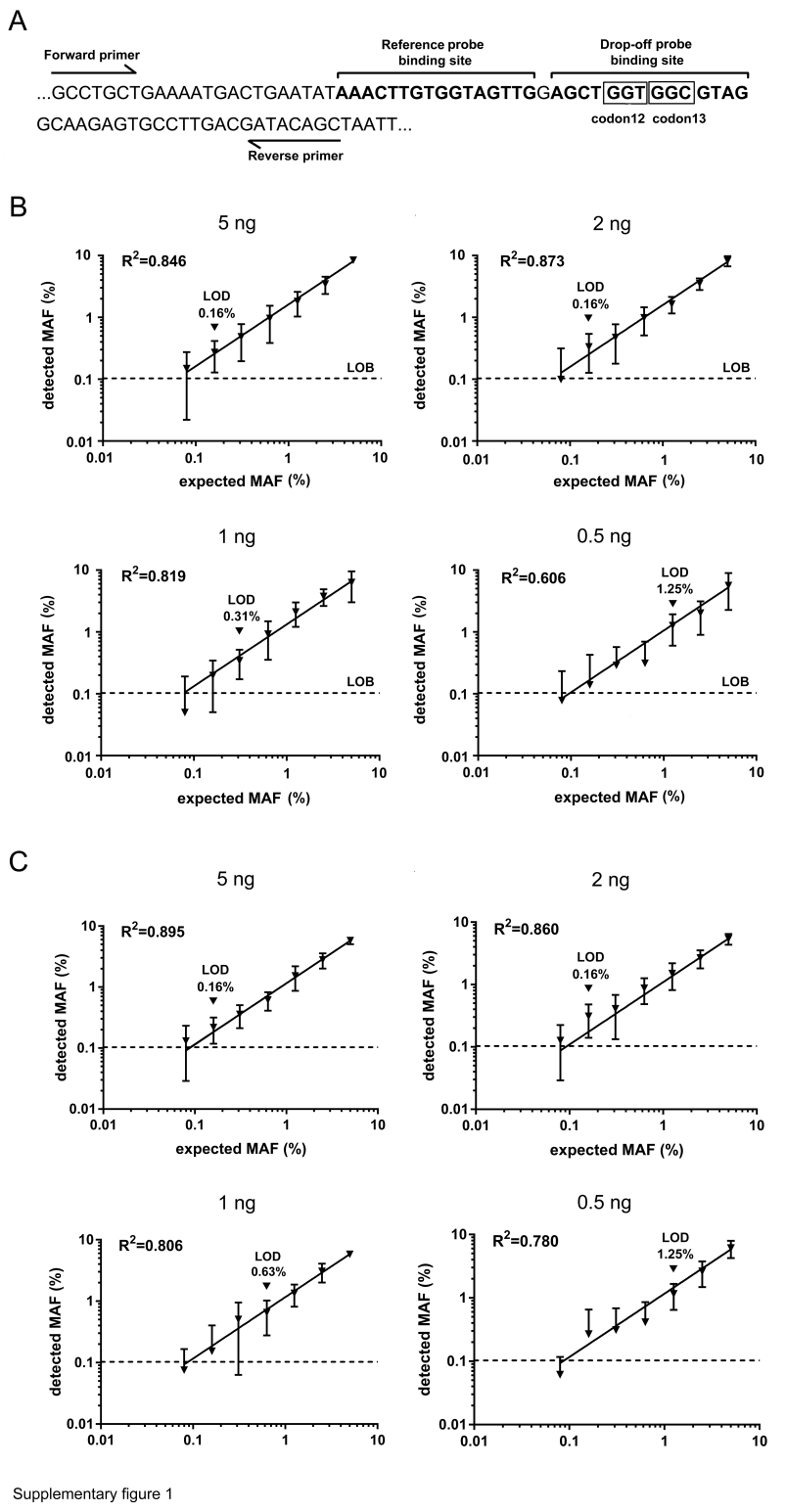
**

**Supplementary Figure 1.** Drop-off assay design and sensitivity analysis of the ddPCR assay. **(A)** Drop-off assay design on partial sequence of *KRAS* exon 2. **(B)**-**(C)** Detected MAF versus expected MAF in serial dilutions under different quantities of cfDNA template with mutant KRAS G12D (B) and G13D (C), respectively.
